# Exercise Training Alleviates Hypoxia-induced Mitochondrial Dysfunction in the Lymphocytes of Sedentary Males

**DOI:** 10.1038/srep35170

**Published:** 2016-10-12

**Authors:** Hsing-Hua Tsai, Shao-Chiang Chang, Cheng-Hsien Chou, Tzu-Pin Weng, Chih-Chin Hsu, Jong-Shyan Wang

**Affiliations:** 1 Healthy Aging Research Center, Graduate Institute of Rehabilitation Science, Medical Collage, Chang Gung University, Tao-Yuan, Taiwan; 2 Department of Physical Medicine and Rehabilitation, Chang Gung Memorial Hospital, Keelung, Taiwan

## Abstract

This study elucidates how interval and continuous exercise regimens affect the mitochondrial functionality of lymphocytes under hypoxic stress. Sixty healthy sedentary males were randomly assigned to engage in either high-intensity interval training (HIIT, 3 min intervals at 80% and 40% VO_2max_, n = 20) or moderate-intensity continuous training (MICT, sustained 60% VO_2max_, n = 20) for 30 min/day, 5 days/week for 6 weeks or were assigned to a control group that did not receive exercise intervention (n = 20). Lymphocyte phenotypes/mitochondrial functionality under hypoxic exercise (HE, 100 W under 12% O_2_) were determined before and after the various interventions. Before the intervention, HE (i) increased the mobilization of senescent (CD57^+^/CD28^−^) lymphocytes into the blood, (ii) decreased the ATP-linked O_2_ consumption rate (OCR), the reserve capacity of OCR, and the citrate synthase activity in the mitochondria, and (iii) lowered the mitochondrial membrane potential (MP) and elevated the matrix oxidant burden (MOB) of lymphocytes. However, both HIIT and MICT significantly (i) decreased blood senescent lymphocyte counts, (ii) enhanced the mitochondrial OCR with increased citrate synthase and succinate dehydrogenase activities, (iii) increased mitochondrial MP and decreased MOB and (iv) increased the ratio of mitofusin to DRP-1 in lymphocytes after HE. Thus, we concluded that either HIIT or MICT effectively improves lymphocyte mitochondrial functionality by enhancing oxidative phosphorylation and suppressing oxidative damage under hypoxic conditions.

Lymphocyte mitochondria play an essential role in lymphocyte immune function^1^. Severe hypoxia increases the mobilization of senescent lymphocytes and enhances lymphocyte apoptosis by reducing the cellular antioxidant levels^2^,^3^. Elevated oxidative stress following severe hypoxia may disrupt the regulation of mitochondrial fission and/or fusion and subsequently impairs mitochondrial integrity and bioenergetics^4^. Our previous study showed that exercise training under hypoxic conditions decreases senescent T-lymphocyte subsets in the blood, along with decreased levels of oxidative stress and pro-inflammatory cytokine production^5^. However, physical exercise inconsistently enhances and suppresses immunity, depending on the intensity and amount of exercise^6^,^7^. To our knowledge, no clear and comprehensive picture of the distinct effects of various exercise regimens on lymphocyte mitochondrial quality/quantity under hypoxic conditions is available.

High-intensity interval training (HIIT) is a system of organizing cardio-respiratory training that involves repeated bouts of short duration, high-intensity exercise intervals alternating with lower intensity intervals of active recovery^8^,^9^,^10^,^11^,^12^,^13^. At the same workout volume, HIIT is a more effective modality for improving aerobic capacity than traditional moderate-intensity continuous training (MICT) in healthy sedentary individuals^8^,^9^ and in patients with cardiovascular disorders^10^,^11^,^12^,^13^. Warm-up exercise (40% VO_2max_) has been shown to decrease high-intensity exercise (80% VO_2max_)-induced risks of inflammatory thrombosis associated with leukocytes and platelets, which is a form of preconditioning^14^. Recently, we have demonstrated that HIIT (alternating 40% VO_2max_ and 80% VO_2max_) effectively diminishes the hypoxia-induced depressed autophagy and potentiates apoptosis in CD4 lymphocytes compared with MICT (sustained 60%VO_2max_)^9^. Therefore, we hypothesize that HIIT is superior to MICT in increasing the resistance to mitochondrial dysfunction in lymphocytes undergoing hypoxia.

To answer the abovementioned questions, we evaluated how two isovolumic exercise regimens [*i.e.*, HIIT (3 min intervals at 40% and 80% VO_2max_) and MICT (sustained 60% VO_2max_)] for 6 weeks affected (i) phenotypic characteristics, (ii) mitochondrial oxidative phosphorylation and oxidative stress, and (iii) mitochondrial biogenesis and fusion/fission in lymphocytes after hypoxic exercise (HE). The aim of the present study was to establish an effective exercise strategy for improving individual aerobic capacity and simultaneously ameliorating the risk of lymphocyte mitochondrial dysfunction evoked by hypoxic stress.

## Results

### Aerobic fitness

**Figure 1:**
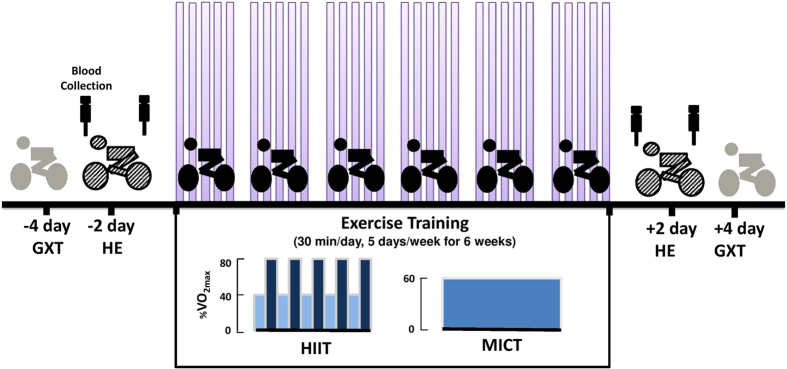
Design and time course of the experiment. Subjects (n = 60) were randomly divided into three groups: control (**CTL**; n = 20), high-intensity interval training (**HIIT**, n = 20), and moderate-intensity continuous training (**MICT**, n = 20). Exercise trained on a bicycle ergometer for five cycles 3-min at 80% of VO_2max_ interspersed with a 3-min active recovery at 40% of VO_2max_ (**HIIT**), or continuous at 60% of VO_2max_ (**MICT**) for 30 min/day, 5 days/week, for 6 weeks. CTL group did not received exercise intervention. Each subject had to perform 1) graded exercise tests (**GXT**) 4 days before and 4 days after the intervention, 2) hypoxic exercise test (**HE**) on the second day before and on the second day after the intervention. At rest and immediately after the HE test, blood samples were collected.

The design and time courses of both HIIT and MICT are shown in Fig. 1. Anthropometric variables did not significantly differ among the three groups at the beginning of the study (Table 1). At 6 weeks, both HIIT and MICT lowered heart rate (HR) and systolic blood pressure (SBP) at rest and increased the work-rate, exercise time, minute ventilation (V_E_), O_2_ consumption (VO_2_), and CO_2_ production (VCO_2_) at the ventilatory threshold and peak exercise performance (Table 1, *P* < 0.05). Moreover, HIIT had a stronger effect on pulmonary ventilation and aerobic capacity than did MICT (Table 1, *P* < 0.05). However, control subjects that did not receive exercise intervention (CTL) for 6 weeks showed no changes in these cardiopulmonary responses to a graded exercise test (GXT) (Table 1).

### Lymphocyte phenotypes

**Figure 2:**
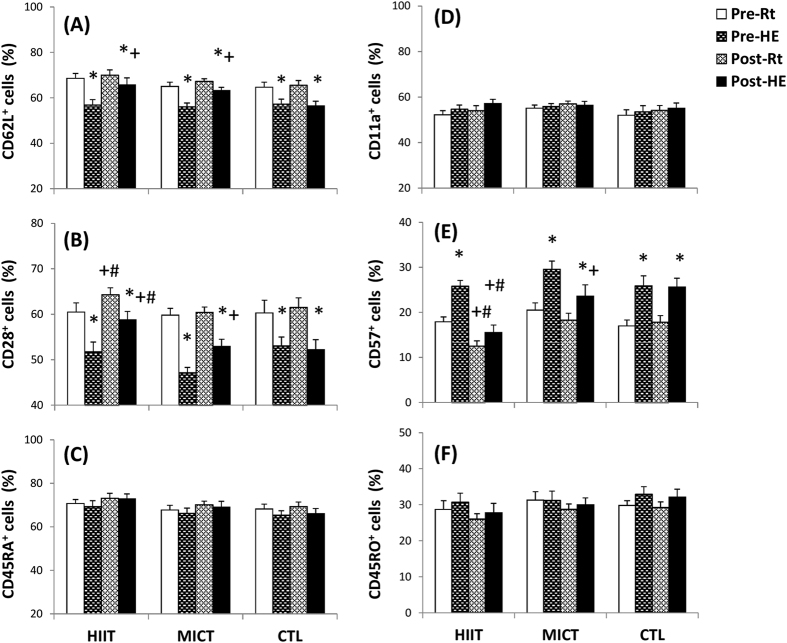
The effects of interval and continuous exercise regimens on lymphocyte phenotypes. **HIIT**, high-intensity interval training group; **MICT**, moderate-intensity continuous training group; **CTL**, control group; **Pre**, pre-intervention; **Post**, post-intervention; **Rt**, resting; **HE**, hypoxic (12%O_2_) exercise test. *P < 0.05, **Rt** vs. **HE**; ^+^*P* < 0.05, **Pre** vs. **Post**; ^#^*P* < 0.05, **AIT** vs. **MCT**. Values were mean ± SE.

Acute hypoxic exercise (HE) significantly increased the blood lymphocyte count, whereas both HIIT and MICT diminished the HE-induced lymphocyte increase (data not shown). Before exercise training, acute HE decreased the percentages of CD62L^+^ (Fig. 2A, *P* < 0.05) and CD28^+^ (Fig. 2B, *P* < 0.05) lymphocytes and increased the percentage of CD57^+^ (Fig. 2E, *P* < 0.05) lymphocytes in the blood. After the 6 week intervention, HIIT increased the percentage of CD28^+^ (%CD28^+^) lymphocytes (Fig. 2B, *P* < 0.05) and decreased the %CD57^+^ lymphocytes (Fig. 2E, *P* < 0.05) at rest and after HE, whereas MICT only increased the %CD28^+^ lymphocytes (Fig. 2B, *P* < 0.05) and decreased the %CD57^+^ lymphocytes (Fig. 2E, *P* < 0.05) at rest. However, neither HIIT nor MICT changed the %CD11a^+^ (Fig. 2D), %CD45RA^+^ (Fig. 2C), or %CD45RO^+^ (Fig. 2F) lymphocytes at rest and after HE. Additionally, there were no significant changes in resting and HE-induced mobilization of various lymphocyte subsets after 6 weeks of CTL (Fig. 2A,F).

### Mitochondrial content, membrane potential (MP), and matrix oxidant burden (MOB) in lymphocytes

**Figure 3:**
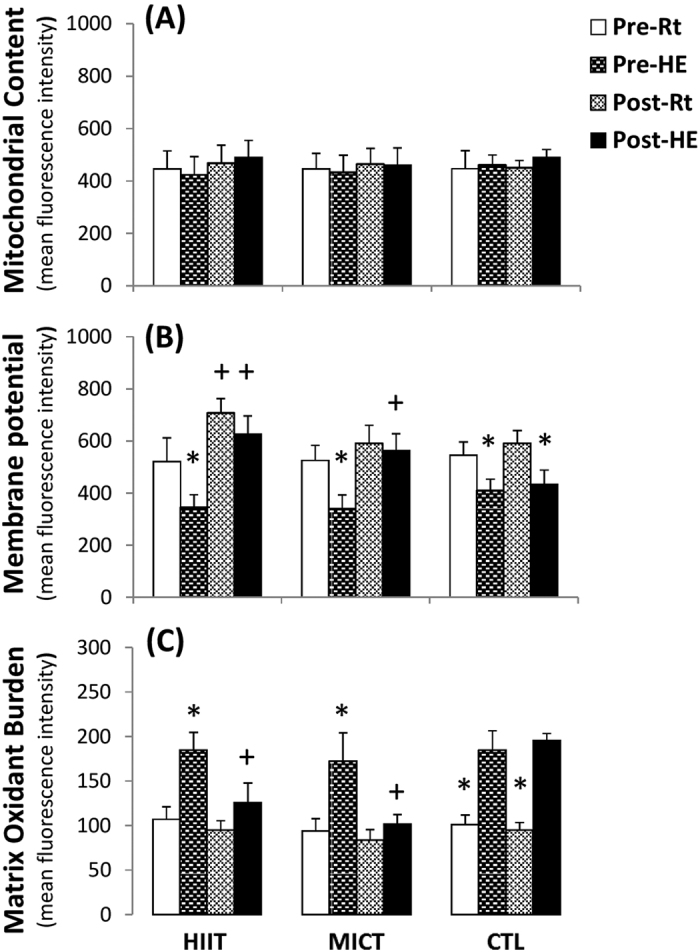
Effects of interval and continuous exercise regimens on (**A**) mitochondrial content, (**B**) mitochondrial membrane potential, and (**C**) matrix oxidant burden in lymphocytes. **HIIT**, high-intensity interval training group; **MICT**, moderate-intensity continuous training group; **CTL**, control group; **Pre**, pre-intervention; **Post**, post-intervention; **Rt**, resting; **HE**, hypoxic (12%O_2_) exercise test. *P < 0.05, **Rt** vs. **HE**; ^+^*P* < 0.05, **Pre** vs. **Post**. Values were mean ± SE.

Although no changes were observed in the mitochondrial count (Fig. 3A), the acute HE decreased the mitochondrial MP (Fig. 3B, *P* < 0.05) and elevated the MOB (Fig. 3C, *P* < 0.05) in lymphocytes. After 6 weeks of the intervention, HIIT increased the mitochondrial MP of lymphocytes at rest (Fig. 3B, *P* < 0.05), whereas both HIIT and MICT inhibited the HE-mediated decreased mitochondrial MP (Fig. 3B, *P* < 0.05) and enhanced the MOB (Fig. 3C, *P* < 0.05) in lymphocytes. However, no significant changes in the mitochondrial count, MP, and MOB of lymphocytes were observed after CTL for 6 weeks (Fig. 3A–C).

### Mitochondrial respiration of lymphocytes

**Figure 4:**
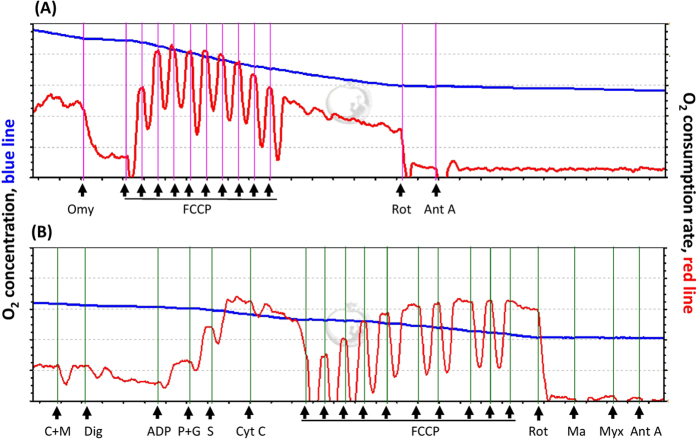
Graph showing measurement of mitochondrial O_2_ consumption rate (OCR) in lymphocytes using a high-resolution respirometry (Oroboros O2K). (**A**), the OCR protocol in intact lymphocytes and (**B**), the OCR [substrate, uncoupler, inhibitor titration (SUIT)] protocol in permeabilized lymphocytes. **Omy**, oligomycin; **FCCP**, carbonyl cyanide-p-trifluoromethoxyphenylhydrazone; **Rot**, rotenone; **Ant A**, antimycin A ; **M**, malate; **C**, palmitoyl-DL carnitine-HCl; **Dig**, digitonin; **ADP**, adenosine diphosphate; **P**, pyruvate; **G**, glutamate; **S**, succinate; **Cyst C**, cytochrome c; **Ma**, malonic acid; **Myx**, myxothiazol.

**Figure 5:**
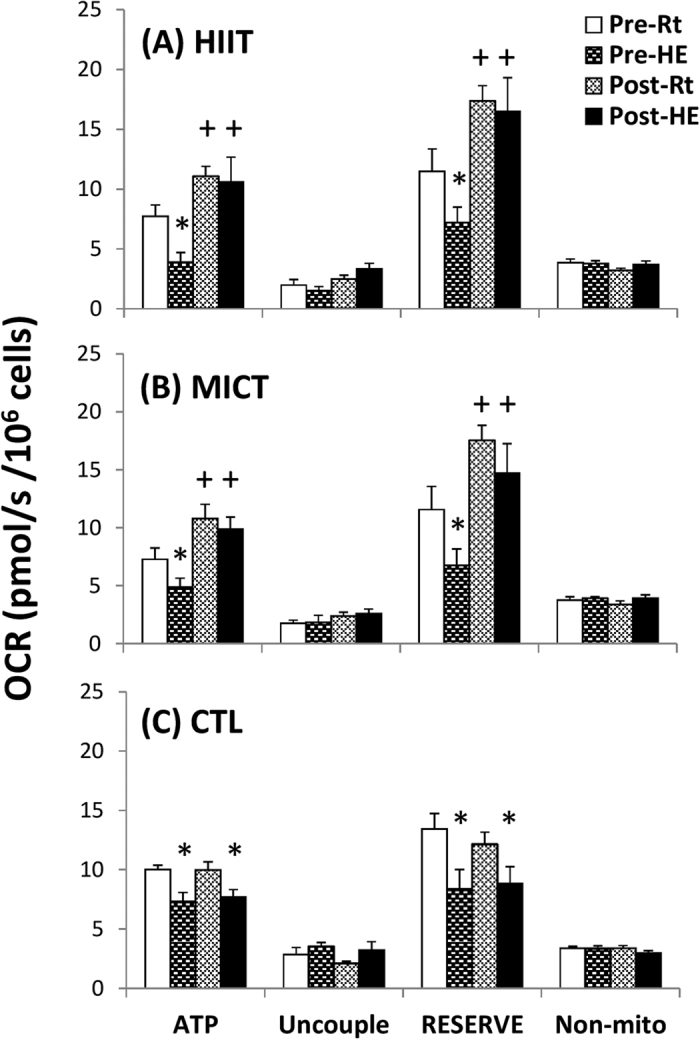
Effects of interval and continuous exercise regimens on mitochondrial O_2_ consumption rate (OCR) in the intact lymphocytes. **HIIT**, high-intensity interval training group; **MICT**, moderate-intensity continuous training group; **CTL**, control group; **Pre**, pre-intervention; **Post**, post-intervention; **Rt**, resting; **HE**, hypoxic (12%O_2_) exercise test; **ATP**, ATP-linked OCR; **Uncouple**, uncouple OCR; **RESERVE**, the reserve capacity of OCR; **Non-mito**, Non-mitochondrial OCR. *P < 0.05, **Rt** vs. **HE**; ^+^*P* < 0.05, **Pre** vs. **Post**. Values were mean ± SE.

**Figure 6:**
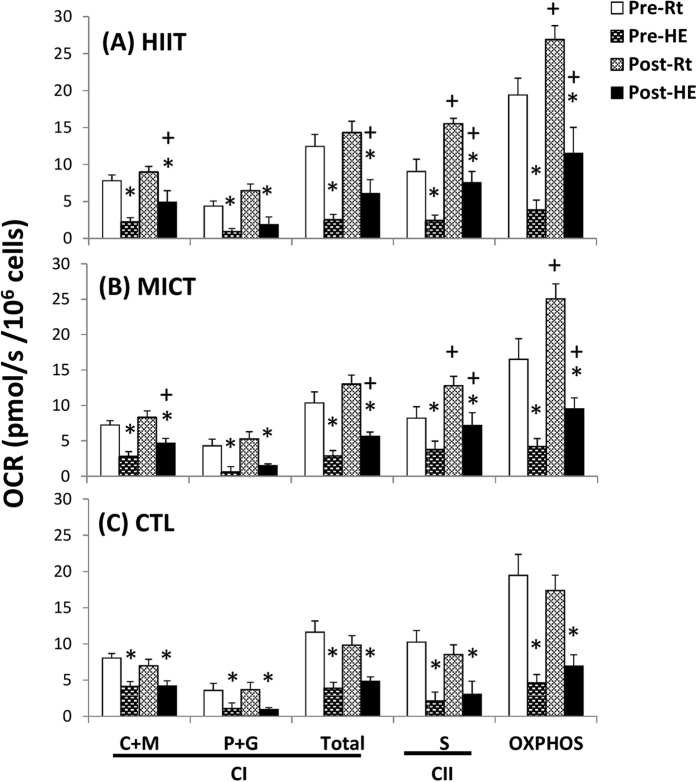
Effects of interval and continuous exercise regimens on mitochondrial O_2_ consumption rate (OCR) in the permeabilized lymphocytes. **HIIT**, high-intensity interval training group; **MICT**, moderate-intensity continuous training group; **CTL**, control group; **Pre**, pre-intervention; **Post**, post-intervention; **Rt**, resting; **HE**, hypoxic (12%O_2_) exercise test; **M**, malate; **C**, palmitoyl-DL carnitine-HCl; **P**, pyruvate; **G**, glutamate; **S**, succinate; **OXPHOS**, capacity of oxidative phosphorylation; **CI**, Complex I respiration; **CII**, Complex I respiration. *P < 0.05, **Rt** vs. **HE**; ^+^*P* < 0.05, **Pre** vs. **Post**. Values were mean ± SE.

Figure 4A,B show analysis of mitochondrial O_2_ consumption rates (OCRs) in the intact and permeabilized lymphocytes, respectively, using high-resolution respirometry (Oroboros O2K). An acute bout of 12% O_2_ exercise significantly decreased ATP-linked OCR and the reserve capacity of OCR in intact lymphocytes (Fig. 5A–C, *P* < 0.05). The acute bout of 12% O_2_ exercise also suppressed the palmitoyl carnitine plus malate (C+M)-, pyruvate plus glutamate (P+G)-, and succinate (S)-mediated OCRs in permeabilized lymphocytes (Fig. 6A–C, *P* < 0.05). After 6 weeks, both HIIT and MICT significantly increased the ATP-linked OCR and elevated the reserve capacity of OCR in intact lymphocytes at rest and after HE (Fig. 5A,B, *P* < 0.05). Moreover, the two exercise regimens enhanced the succinate-mediated OCR and the capacity for oxidative phosphorylation (OXPHO) at rest and diminished the HE-induced depressed C+M-, P+G-, and S-mediated OCRs and OXPHO capacity in lymphocytes (Fig. 6A,B, *P* < 0.05). Additionally, there were no significant changes at rest and in the HE-depressed mitochondrial respiration of lymphocytes after 6 weeks of CTL (Figs 5 and 6).

### Enzyme activities of glycolysis and the Krebs cycle in lymphocytes

Prior to exercise training, acute HE increased the lactate dehydrogenase (LDH) and glutamate dehydrogenase (GDH) activities but decreased the citrate synthase (CS) activity in lymphocytes (Table 2, *P* < 0.05). After 6 weeks of the intervention, neither HIIT nor MICT decreased the HE-induced increased LDH/decreased CS activities of the lymphocytes (Table 2). Moreover, the two exercise regimens significantly enhanced the lymphocyte SDH activity at rest and after HE (Table 2, *P* < 0.05). However, various glycolytic enzyme activities and Krebs cycle enzyme activities in lymphocytes at rest or after HE remained unchanged after 6 weeks of CTL (Table 2).

### Mitochondrial biogenesis and fusion/fission in lymphocytes

Acute HE did not change the levels of phospho-SIRT, PCG-1α, NRF-1, and Tfam and the ratio of Complex IV to II, despite modestly increasing phospho-AMPK in lymphocytes (Table 3). However, no significant changes in these mitochondrial biogenetic variables of lymphocytes occurred after 6 week interventions with HIIT, MICT, and CTL (Table 3).

**Figure 7:**
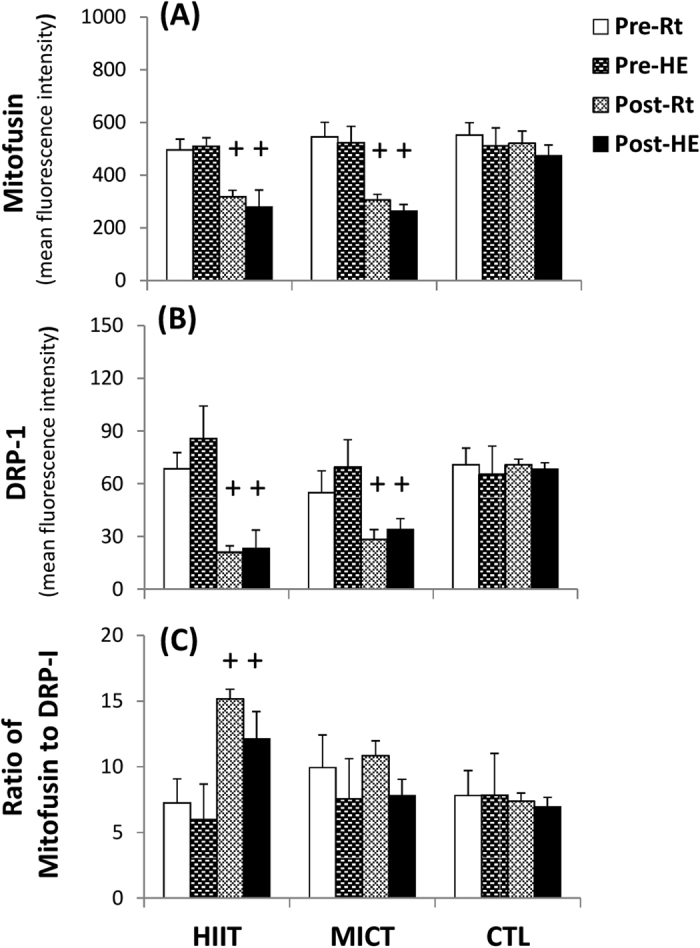
Effects of interval and continuous exercise regimens on mitochondrial fusion and fission in lymphocytes. **HIIT**, high-intensity interval training group; **MICT**, moderate-intensity continuous training group; **CTL**, control group; **Pre**, pre-intervention; **Post**, post-intervention; **Rt**, resting; **HE**, hypoxic (12%O_2_) exercise test. *P < 0.05, **Rt** vs. **HE**; ^+^*P*< 0.05, **Pre** vs. **Post**. Values were mean ± SE.

The mitofusin and DRP-1 levels in lymphocytes were unchanged in response to HE (Fig. 7A–C). Despite the decrease in mitofusin and DRP-1 (Fig. 7A,B, *P* < 0.05), both HIIT and MICT significantly increased the ratio of mitofusin to DRP-1 in lymphocytes at rest and after HE (Fig. 7C, *P* < 0.05). However, CTL for 6 weeks did not change the resting and HE-related mitofusin and DRP-1 contents and the mitofusin/DRP-1 ratio in lymphocytes (Fig. 7A–C).

### Plasma norepinephrine and epinephrine concentrations

At the beginning of the study, acute HE increased both epinephrine and norepinephrine levels in the plasma. After 6 weeks of the intervention, HIIT significantly lowered plasma epinephrine and norepinephrine levels at rest (Table 3, *P* < 0.05), whereas both HIIT and MICT decreased the increased release of epinephrine and norepinephrine caused by HE (Table 4, *P* < 0.05). However, no significant changes in resting and HE-increased plasma catecholamine levels were observed after CTL for 6 weeks (Table 4).

## Discussion

This study clearly showed that HIIT is superior to MICT in enhancing aerobic capacity by increasing pulmonary ventilation and tissue O_2_ utilization at peak performance. Notably, this study is the first to demonstrate that HIIT, but not MICT, lowers senescent lymphocyte distribution and enhances lymphocyte mitochondrial function at rest. However, both HIIT and MICT substantially increase the resistance to senescent lymphocyte mobilization and mitochondrial dysfunction caused by hypoxic stress.

### Phenotypic characteristics in lymphocytes

CD57-positive lymphocytes have shortened telomeres that can no longer enter the cell cycle and are associated with age-related dysfunction of the immune system^15^. Additionally, the CD28-negative lymphocytes also exhibit decreased antigen receptor diversity, defective antigen-induced proliferation, and a shorter replicative lifespan^16^. The decrease in senescent (CD57^+^/CD28^−^) lymphocyte subsets caused by HIIT may enhance the capacity of clonal expansion in lymphocytes. Although MICT did not alter the phenotypic characteristics of lymphocytes at rest, both HIIT and MICT significantly diminished the HE-mediated senescent (CD57^+^/CD28^−^) lymphocyte release. Therefore, the two exercise regimens may minimize HE-induced lymphocyte dysfunction, at least partially, by decreasing mobilization of senescent lymphocytes into the peripheral blood.

Increased oxidative stress and pro-inflammatory cytokines have been shown to down-regulate CD28 expression on leukocytes^17^,^18^. Additionally, norepinephrine preferentially modulates the function of immune cells by inducing inflammatory cytokine production and reducing immune cell expansion^19^. A clinical investigation has demonstrated that HIIT decreases plasma myeloperoxidase or/and interleukin-6 levels in patients with heart failure^10^. Therefore, we hypothesized that HIIT decreases the percentage of senescent lymphocyte subsets in the blood, which may be associated with decreased oxidative stress/pro-inflammatory status and decreased catecholamine levels in blood.

### Mitochondrial oxidative phosphorylation in lymphocytes

Mitochondria are highly sensitive to hypoxic stress and respond dynamically to changes in their cellular microenvironment^20^. The present study showed that HE increased MMT and decreased MOB in lymphocytes, which may reflect mitochondrial oxidative damage of lymphocytes caused by hypoxic stress. In intact cells, the HE-induced decrease in ATP-linked OCR and reserve capacity of OCR may be associated with modified activities of mitochondrial enzymes and/or an impeded the flow of electrons, thereby decreasing oxidative phosphorylation after HE. Acute 12% O_2_ exercise significantly decreased CS activity and increased the LDH activity, thus suggesting that HE shifts lymphocyte metabolic pathways from mitochondrial respiration to glycolysis^21^. Additionally, HE increased the GDH activity of lymphocytes, which may subsequently enhance glutamate utilization for amino acid metabolism^22^. In permeabilized cells, HE substantially depressed the Complex I- and II-related OCRs and OXPHOS capacity in lymphocytes in a substrate-rich environment. The results indicate that HE globally decreases mitochondrial substrate availability and impairs mitochondrial bioenergetics and/or integrity in lymphocytes.

The enhanced OCR indicates a greater efficiency of lymphocyte mitochondria due to increased Complex protein activities of the electronic transfer system (ETS), elevated ATP production rate, or heightened levels of NADH and FADH_2_^23^. Hence, increased resistance to HE-depressed lymphocyte mitochondrial OCR after HIIT or MICT may improve the flexibility during hypoxia-induced lymphocyte dysfunction. Intriguingly, the two exercise regimens significantly enhanced lymphocyte SDH activity and Complex II respiration at rest and after HE. Recent studies have indicated that hypoxia or ischemia results in accumulation of intracellular succinate levels, thus leading to elevated production of mitochondrial reactive oxygen species (ROS), and subsequently triggers cell apoptosis^24^. The pro-inflammatory status may also induce succinate accumulation by decreasing SDH activity^25^. Therefore, the increased SDH activity and Complex II respiration of lymphocytes in both training groups may quickly eliminate succinate, thereby further retarding the inflammatory signaling and ROS production under hypoxic stress^25^.

### Mitochondrial biogenesis and fusion/fission in lymphocytes

Early studies on exercise intervention predominantly focused on mitochondrial functionality in skeletal muscles^26^. An acute bout of exercise promotes transcriptional or post-translational regulation of PGC-1α, whereas chronic exercise increases the rates of muscular mtDNA gene expression by upregulating Tfam in skeletal muscles^26^. In the present study, no significant changes in resting and HE-related lymphocyte biogenetic parameters, such as SIRT-1, PCG-1α, NRF-1, Tfam, and the Complex IV/II ratio, were observed after 6 weeks of HIIT or MICT. Lymphocytes are normally in a quiescent state and rely on metabolic pathway shifts to meet their energetic demands upon activation^27^. Accordingly, we propose that lymphocyte metabolic adaptation induced by exercise training may be associated with improved ETS efficiency/capacity rather than modulated mitochondrial biogenesis in lymphocytes.

In mitochondrial dynamics, endurance exercise training depresses DRP-1 activation in insulin-resistant human skeletal muscles^28^, whereas HIIT increases mitofusin and decreases DRP-1 levels in the skeletal muscles of rats with post-myocardial infarction^29^. This study was the first to explore the effects of chronic exercise on the balance between mitochondrial fusion and fission in lymphocytes. Despite a modest decrease in mitofusin levels, both HIIT and MICT substantially down-regulated DRP-1 and increased the ratio of mitofusin to DRP-1 in lymphocytes. These results indicate that the two exercise regimens tend to shift lymphocyte mitochondria toward fusion and subsequently improve the bioenergetic efficiency of lymphocytes.

### Conclusions

In this study, 6 weeks of HIIT resulted in a higher aerobic capacity than the MICT regimen. Acute 12% O_2_ exercise increased the mobilization of senescent lymphocytes into the peripheral blood and resulted in elevated oxidative stress and decreased oxidative phosphorylation in lymphocyte mitochondria. Although no changes were found in mitochondrial biogenesis, both HIIT and MICT regimens effectively shifted lymphocyte mitochondria toward fusion, attenuated the HE-induced release of senescent subsets and lowered the mitochondrial OXPHOS capacity in lymphocytes. Therefore, the two exercise regimens effectively improve lymphocyte bioenergetics, possibly by enhancing mitochondrial quality rather than quantity in lymphocytes. These experimental findings may facilitate the identification of effective exercise training regimens to increase aerobic capacity and minimize mitochondrial dysfunction in lymphocytes under hypoxic conditions.

## Materials and Methods

### Subjects

The study was in accordance with the Declaration of Helsinki and approved by the Chang Gung Memorial Hospital Institutional Review Board, Taiwan. A total of 60 sedentary males who were non-smokers, did not use medications or vitamins, and were free of any cardiopulmonary/hematological risks were recruited from Chang Gung University, Taiwan. No subjects had engaged in any regular physical activity (exercise frequency ≤ once weekly, duration <20 min) or had been exposed to high altitudes (≥altitude of 3000 m) for at least 1 year before the experiment. All subjects provided informed consent after the experimental procedures were explained. These subjects were randomly divided into three groups: the HIIT (n = 20), MICT (n = 20) and CTL (n = 20) groups. Moreover, all subjects arrived at the testing center at 9:00 AM to eliminate any possible circadian effects. Participants were instructed to fast for at least 8 hours and to refrain from strenuous physical exercise for at least 48 hours before sampling.

### Training protocols

Both the HIIT and MICT groups performed exercise regimens on a stationary bicycle ergometer 5 times a week for 6 weeks (Fig. 1). For comparison, CTL participants did not any undergo any exercise but were carefully monitored, and we recorded information on their physical activity and nutritional intake for 6 weeks^8^,^9^. HIIT subjects warmed up for 3 min at 30% of maximal O_2_ consumption (VO_2max_) before starting five exercise cycles, each lasting 3 min at 80% of VO_2max_ interspersed with a 3 min active recovery period at 40% of VO_2max_. The exercise session was terminated with a 3 min cool-down period at 30% of VO_2max_. The MICT group had the same warm-up and cool-down protocols as the HIIT group, except that the training period was 30 min at 60% of VO_2max_^8^,^9^. The two exercise protocols were isovolumic with the same exercise duration [i.e., HIIT exercise volume: 6 min (40%VO_2max_ + 80% VO_2max_) × 5 cycles) = MICT exercise volume: 30 min (60% VO_2max_)]. Each subject used a HR monitor (Tango, SunTech Medical) to obtain the assigned intensity of exercise. The work-rate of the bicycle ergometer was adjusted continuously to ensure that the intensity of exercise matched the target HR throughout the training period.

All subjects recorded their daily activities and nutritional intake throughout the experiment using the International Physical Activity Questionnaire Short Form^30^ and the Written Diet Record^31^, respectively. The participants were instructed to refrain from extra regular exercise until the end of the study. Moreover, the participant compliance rates for the three interventions were 100%. All subjects completed the exercise intervention and/or tests at the beginning of the present study and after 6 weeks in the three groups.

### Graded exercise test (GXT)

Subjects performed a GXT on a bicycle ergometer (Corival 400, Lode) to assess their aerobic capacity 4 days before and 4 days after the 6 week interventions^8^,^9^. The GXT was composed of 2 min of unloaded pedaling followed by a continuous increase in work-rate of 30 W per 3 min until exhaustion (*i.e.*, VO_2max_). The V_E_, VO_2_, and VCO_2_ were measured breath by breath with a computer-based system (MasterScreen CPX, Cardinal-health Germany). The defined VO_2max_ was required to achieve the following 3 of 4 criteria: (i) the level of VO_2_ increased less than 2 mL/kg/min over at least 2 min; (ii) HR exceeded its predicted maximum; (iii) the respiratory exchange ratio exceeded 1.2, and (iv) the venous lactate concentration exceeded 8 mM, consistently with the guidelines of the American College of Sports Medicine for exercise testing (American College of Sports Medicine, 2010)^32^.

### Hypoxic exercise (HE) test and blood collection

Each subject performed the HE test on the 2^nd^ day before the intervention and on the 2^nd^ day after the intervention in an air-conditioned normobaric hypoxia chamber (Colorado Mountain Room, Boulder, CO) as described in our previous studies^8^,^9^. The hypoxia chamber was maintained at a temperature of 22 +/− 0.5 °C with a relative humidity of 60 +/− 5%; a CO_2_ scrubber eliminated CO_2_ in the air (<3,500 ppm). The HE test on the bicycle ergometer required 50 W of warm-up for 3 min, an increase in the work-rate to 100 W with continuous exercise for 30 min, and then recovery to 50 W of a cool-down period for 3 min. During the test, the O_2_ concentration was set to 12%, which corresponds to an altitude of 4,460 m^8^,^9^.

At rest and immediately after the HE test, 40 ml blood samples were collected from an antecubital vein using a clean venipuncture (20 gauge needle). The first 2 ml of blood was discarded, and the remaining blood was used to measure hematological parameters. Blood cells were counted using a Sysmax SF-3000 cell counter (GMI, Inc., Ramsey, MN).

### Lymphocyte isolation

Blood samples were transferred to polypropylene tubes containing sodium citrate (3.8 g/dl: 1 vol. to 9 vol. of blood) (Sigma, St-Louis, MI). Lymphocytes were isolated by density-gradient centrifugation in Lymphoprep tubes (Nycomed). Isolated cells were then washed three times in Roswell Park Memorial Institute (RPMI) medium (Gibco, Invitrogen; pH 7.4). The number of lymphocytes was adjusted to 2×10^6^ cells/ml with RPMI medium. The analysis of lymphocyte functions was completed within 2 hours after cell purification^9^.

### Blood lymphocyte phenotypic characteristics

The lymphocyte suspensions (2×10^6^ cells/ml) were incubated with saturating concentrations (10 μg/mL) of monoclonal anti-human CD57 (eBioscience), CD28 (eBioscience), CD62L (eBioscience), CD11a (eBioscience), CD45RA (eBioscience), or CD45RO (eBioscience) conjugated to fluorescein isothiocyanate (FITC) or anti-rabbit IgG (eBioscience) control antibody conjugated to FITC in the dark for 30 min at 4 °C. Lymphocytes treated with the control antibody were utilized to correct for background fluorescence. After fixation with 2% formaldehyde in phosphate-buffered saline solution (PBS), the fluorescence recorded from 10,000 events representing the lymphocytes was calculated using a single-color FACScan flow cytometer (Becton Dickinson), as described in our previous study^5^.

### Mitochondrial content, MP, and MOB in lymphocytes

The relative quantification of lymphocyte mitochondrial-localized dyes using the single-color flow cytometric analysis allows for the sensitive measurement of a variety of mitochondrial parameters, including mitochondrial content, mitochondrial MP, and matrix oxidant burden (MOB), as described in a previous study^33^. The lymphocyte suspensions (2×10^6^ cells/ml) were incubated with MitoTracker Green FM (200 nM) (Invitrogen), TMRE (20 nM) (Invitrogen), or MitoSOX Red (6.6 μM) (Invitrogen) in the dark for 30 min at 4 °C. Then, the lymphocytes were gated separately from other blood cells on the basis of forward/sideways scatter, and the mean fluorescence intensity from 10,000 events representing the lymphocytes was calculated using a FACScan flow cytometer (Becton Dickinson).

### Mitochondrial respiration of intact lymphocytes

The mitochondrial O_2_ consumption of lymphocytes (2×10^6^ cells/ml) in RPMI 1460 medium was measured using high-resolution respirometry (Oroboros O2K). Mitochondrial respiration coupled with ATP production (ATP-linked O_2_ consumption rate, ATP-linked OCR) was measured on the basis of the decrease in O_2_ consumption after the addition of oligomycin (0.2 μg/ml), an inhibitor of ATP synthase. The remaining rate of mitochondrial respiration represents a proton leak that uncouples oxidative phosphorylation from the electron transport system (ETS). The total O_2_ consumption of lymphocytes was measured at baseline and after the addition of the uncoupling agent carbonyl cyanide-p-trifluoromethoxyphenylhydrazone (FCCP; 2 μM) to induce maximal O_2_ consumption. The difference between the basal and maximal respiration is called the reserve capacity of OCR. Non-mitochondrial respiration was quantified by inhibiting mitochondrial respiration through the addition of rotenone (1 μM) and antimycin A (1 μM) (Fig. 4A)^26^.

### Mitochondrial respiration of permeabilized lymphocytes

A substrate, uncoupler, inhibitor titration (SUIT) protocol was used to establish the respiratory capacity with electron flow through fatty acid oxidation and both mitochondria Complex I and Complex II separately as well as convergent electron input *via* the Q-junction. Oxygen consumption rates were balanced for two min at each stage of data collection.

Next, 2.1 mL of mitochondria medium MiR05 (EGTA 0.5 mM, MgCl_2 _· 6H_2_O 3 mM, lactobionic acid 60 mM, taurine 20 mM, KH_2_PO_4_ 10 mM, HEPES 20 mM, D-sucrose 110 mM, and BSA 1 g/L, pH = 7.1) was added in the O2K chamber (Oroboros O2K). The temperature in the chamber was 37 °C. After the temperature was stabilized, the chamber was closed by fully inserting the stopper and extruding all gas bubbles. We siphoned off excess liquid and started the air calibration to calibrate the oxygen concentration in the medium. We injected 4 × 10^6^ cells with 100 mL PBS in the O2K chamber after air calibration. The final cell density in the medium was 2 × 10^6^ cells/ml.

We started data acquisition and waited for 10 min until the oxygen flux stabilized. The oxygen consumption in this state was the routine respiration from endogenous substrates in cells. After the routine respiration was measured, the titration started with a concomitant addition of malate (2 mM) and palmitoyl-DL-carnitine-HCl (20 μM). The plasma membrane was permeabilized by a slow titration of digitonin (20 μg). The respiration decreased in the absence of ADP. The protons leaked through the mitochondrial inner membrane and led to the leak respiration state. The oxygen consumption by fatty acid oxidation was evaluated by addition of 1 mM ADP (Calbiochem). The OXPHOS capacity of Complex I, driven by NADH-related substrates, was acquired through the addition of pyruvate (5 mM) and glutamate (10 mM). Sequentially, 10 mM succinate was added to induce maximal OXPHOS capacity with convergent input through both Complex I and Complex II. A cytochrome c (10 μM) test was applied to evaluate whether the outer mitochondrial membrane was intact. The maximal convergent capacity of the ETS was subsequently obtained by FCCP titration (0.75 μM/steps).

Finally, the inhibitors for Complex I, II and III (0.1 μM rotenone, 5 mM malonic acid, and 0.5 μM myxothiazol/2.5 μM antimycin A) were progressively added to suppress the ETS in lymphocytes. The residual oxygen consumption was not due to mitochondrial respiration (Fig. 4B). All chemicals were purchased from Sigma-Aldrich (St Louis, MO, USA) if not stated otherwise.

### Enzyme activities of glycolysis and the Krebs cycle in lymphocytes

The activities of hexokinase (HK) (Sigma), pyruvate kinase (PK) (Sigma), pyruvate dehydrogenase (PDH) (BioVision), and lactate dehydrogenase (LDH) (Sigma) in glycolysis and the activities of citrate synthase (CS) (BioVision), glutamate dehydrogenase (GDH) (Sigma), and succinate dehydrogenase (SDH) (BioVision) in the Krebs cycle of lymphocytes (2×10^6^ cells/ml) were measured with commercially available colorimetric kits according to the manufacturer’s instructions.

### Mitochondrial biogenesis and fusion/fission in lymphocytes

Isolated lymphocytes (2×10^6^ cells/ml) were permeabilized using a commercial permeabilization washing buffer (eBioscience) and then incubated at 4 °C for 30 min in the dark with a saturation concentration (10 μg/mL) of monoclonal anti-human phospho-AMPK (Abcam), SIRT1 (Abcam), PGC1-α (Abcam), NRF1 (Abcam), TFAM (Abcam), UCP2 (Santa Crus Biotechnology), Mfn1 (Abcam), or Drp1 (Abcam) antibody conjugated with FITC or anti-rabbit IgG (eBioscience) control antibody conjugated with FITC in the dark for 30 min at 4 °C. Lymphocytes treated with the control antibody were utilized to correct for background fluorescence. After fixation with 2% formaldehyde in PBS, the fluorescence from 10,000 events representing the lymphocytes was calculated using a FACScan flow cytometer (Becton Dickinson). Additionally, lymphocyte mitochondrial biogenesis, indicated by a ratio of Complex IV to II, was measured with a commercially available flow cytometry kit (MitoBiogenesis™, Abcam), according to the manufacturer’s instructions.

### Plasma norepinephrine and epinephrine concentrations

From all subjects, 5 ml of blood was obtained, placed in a cold centrifuge tube containing EDTA (final concentration, 4 mM) (Sigma Chemical Co.), and immediately centrifuged at 3,000*g* for 10 min at 4 °C. The plasma samples were stored at −80 °C until the assay. Plasma norepinephrine and epinephrine (Labor Diagnostika Nord GmbH & Co.) concentrations were quantified by commercially available ELISA kits.

### Statistical analysis

The results are expressed as the mean ± SEM. The statistical software package StatView was used for data analysis. The Kolmogorov-Smirnov goodness-of-fit test was used, and a normal distribution in all variables was observed in the present study. Experimental results were analyzed by 3 (groups) × 4 (time sample points) repeated measures ANOVA and Bonferroni’s post-hoc test to compare lymphocyte phenotypic characteristics, lymphocyte mitochondrial oxidative phosphorylation, oxidative stress, biogenesis, fusion/fission, and plasma catecholamine levels before and immediately after HE at the beginning of the present study and after 6 weeks in various groups. In addition, the comparison of cardiopulmonary fitness during GXT at the beginning of the present study and 6 weeks later in various groups was analyzed by 3 (groups) × 2 (time sample points) repeated measures ANOVA and Bonferroni’s post-hoc test. The criterion for statistical significance was *P* < 0.05.

## Additional Information

**How to cite this article**: Tsai, H.-H. *et al*. Exercise Training Alleviates Hypoxia-induced Mitochondrial Dysfunction in the Lymphocytes of Sedentary Males. *Sci. Rep.* **6**, 35170; doi: 10.1038/srep35170 (2016).

## Figures and Tables

**Table 1:** The effects of interval and continuous exercise regimens on exercise performance.

	HIIT		MICT		CTL	
	Pre	Post	Pre	Post	Pre	Post
Anthropometric parameters						
Age (year)	23.0 ± 1.7	—	22.1 ± 0.9	—	22.5 ± 1.3	—
Height (cm)	172.3 ± 1.1	—	174.2 ± 1.8	—	173.6 ± 1.9	—
Weight (kg)	66.9 ± 1.3	66.6 ± 1.2	68.5 ± 2.5	68.0 ± 2.3	67.8 ± 2.1	68.0 ± 2.2
BMI (kg/m^2^)	22.5 ± 0.6	22.4 ± 0.5	22.6 ± 0.7	22.4 ± 0.6	22.7 ± 0.6	22.8 ± 0.7
HR (beats/min)	72 ± 1	68 ± 2^+^	71 ± 2	67 ± 2^+^	73 ± 2	72 ± 3
SBP (mmHg)	120 ± 3	115 ± 2^+^	121 ± 3	116 ± 3^+^	123 ± 2	122 ± 3
DBP (mmHg)	74 ± 2	72 ± 2	74 ± 3	72 ± 2	75 ± 3	74 ± 3
Ventilation threshold						
Work-rate (watt)	101.2 ± 6.9	167.1 ± 8.9^+#^	97.2 ± 6.8	155.3 ± 9.4^+^	105.0 ± 9.3	119.0 ± 6.9
HR (beats/min)	137 ± 4	157 ± 4^+#^	136 ± 4	150 ± 3^+^	134 ± 3	136 ± 4
Exercise time (min)	11.5 ± 0.9	19.5 ± 1.2^+#^	10.7 ± 0.7	18.2 ± 1.2^+^	12.1 ± 1.2	13.3 ± 0.9
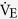 (l/min)	37.6 ± 2.4	56.9 ± 3.7^+#^	34.3 ± 2.2	51.8 ± 3.3^+^	36.4 ± 3.2	38.5 ± 2.1
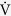 O_2_ (ml/min/kg)	18.1 ± 0.8	28.7 ± 1.9^+#^	18.0 ± 1.4	25.8 ± 1.6^+^	17.3 ± 0.9	18.1 ± 0.8
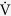 CO_2_ (ml/min/kg)	18.0 ± 0.7	28.8 ± 1.8^+#^	18.1 ± 1.2	25.9 ± 1.9^+^	17.1 ± 0.8	18.2 ± 0.9
Peak exercise performance						
Work-rate (watt)	190 ± 5	240 ± 6^+#^	190 ± 6	220 ± 8^+^	200 ± 8	210 ± 10
HR (beats/min)	195 ± 5	194 ± 2	195 ± 2	196 ± 2	194 ± 2	195 ± 3
Exercise time (min)	25.7 ± 1.2	32.2 ± 0.8^+#^	25.1 ± 1.3	29.8 ± 1.3^+^	25.5 ± 1.3	26.9 ± 1.4
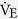 (l/min)	114.8 ± 4.6	133.1 ± 4.0^+#^	106.3 ± 4.7	116.0 ± 4.4^+^	115.9 ± 4.5	114.7 ± 5.9
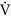 O_2_ (ml/min/kg)	34.0 ± 1.4	40.9 ± 1.8^+#^	33.1 ± 1.2	37.7 ± 1.6^+^	32.2 ± 1.0	35.1 ± 2.1
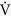 CO_2_ (ml/min/kg)	40.9 ± 1.7	48.7 ± 2.2^+#^	39.9 ± 1.4	45.0 ± 1.9^+^	38.7 ± 1.1	38.8 ± 1.0

Values were mean ± SE. **HIIT**, high-intensity interval training group; **MICT**, moderate-intensity continuous training group; **CTL**, control group; **Pre**, pre-intervention; **Post**, post-intervention; **BMI**, body mass index; 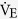, minute ventilation; 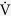
**O**_**2**_, oxygen consumption; 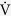
**CO**_**2**_, carbon dioxide production. ^+^*P* < 0.05, **Pre** vs. **Post**; ^#^*P* < 0.05, **HIIT** vs. **MICT**.

**Table 2:** The effects of interval and continuous exercise regimens on enzyme activities of glycolysis and Krebs cycle in lymphocytes.

		HIIT		MICT		CTL	
	Pre	Post	Pre	Post	Pre	Post	
Glycolysis							
Hexokinase activity (nmol/min/10^6^ cells)							
**Rt**	3.75 ± 0.30	3.85 ± 0.21	3.63 ± 0.22	3.62 ± 0.13	4.10 ± 0.21	3.90 ± 0.19	
**HE**	3.60 ± 0.33	4.27 ± 0.22	3.87 ± 0.26	4.23 ± 0.25	4.17 ± 0.23	4.03 ± 0.20	
Pyruvate kinase activity (nmol/min/10^6^ cells)							
**Rt**	56.1 ± 3.4	55.6 ± 2.3	54.6 ± 3.4	57.0 ± 2.7	51.5 ± 4.0	45.2 ± 3.5	
**HE**	51.0 ± 4.5	58.9 ± 3.4	53.6 ± 4.5	60.1 ± 3.4	48.7 ± 3.8	47.9 ± 3.8	
Pyruvate dehydrogenase activity (nmol/min/10^6^ cells)							
**Rt**	0.48 ± 0.02	0.51 ± 0.03	0.50 ± 0.02	0.46 ± 0.02	0.53 ± 0.03	0.47 ± 0.03	
**HE**	0.47 ± 0.03	0.54 ± 0.03	0.54 ± 0.05	0.52 ± 0.03	0.47 ± 0.03	0.50 ± 0.02	
Lactate dehydrogenase activity (nmol/min/10^6^ cells)							
**Rt**	1.35 ± 0.16	1.36 ± 0.11	1.58 ± 0.15	1.52 ± 0.21	1.59 ± 0.11	1.54 ± 0.23	
**HE**	1.92 ± 0.17*	1.57 ± 0.12	2.14 ± 0.32*	1.67 ± 0.09	2.02 ± 0.12*	2.05 ± 0.35*	
Krebs cycle							
Citrate synthase activity (nmol/min/10^6^ cells)							
**Rt**	0.31 ± 0.02	0.31 ± 0.02	0.30 ± 0.02	0.32 ± 0.02	0.30 ± 0.02	0.33 ± 0.03	
**HE**	0.24 ± 0.03*	0.30 ± 0.03	0.23 ± 0.02*	0.30 ± 0.03	0.24 ± 0.02*	0.25 ± 0.02*	
Glutamate dehydrogenase activity (nmol/min/10^6^ cells)							
**Rt**	0.40 ± 0.03	0.43 ± 0.05	0.42 ± 0.03	0.46 ± 0.03	0.42 ± 0.03	0.40 ± 0.02	
**HE**	0.56 ± 0.06*	0.63 ± 0.08*	0.59 ± 0.07*	0.64 ± 0.07*	0.59 ± 0.07*	0.64 ± 0.06*	
Succinate dehydrogenase activity (nmol/min/10^6^ cells)							
**Rt**	0.80 ± 0.15	1.10 ± 0.20^+^	0.77 ± 0.14	1.08 ± 0.14^+^	0.83 ± 0.14	0.85 ± 0.12	
**HE**	0.73 ± 0.11	1.21 ± 0.25^+^	0.74 ± 0.13	1.19 ± 0.11^+^	0.72 ± 0.13	0.91 ± 0.08	

Values were mean ± SE. **HIIT**, high-intensity interval training group; **MICT**, moderate-intensity continuous training group; **CTL**, control group; **Pre**, pre-intervention; **Post**, post-intervention; **Rt**, resting; **HE**, hypoxic exercise test. ^*^P < 0.05, **Rt** vs. **HE**; ^+^*P* < 0.05, **Pre** vs. **Post**.

**Table 3:** The effects of interval and continuous exercise regimens on mitochondrial biogenesis of lymphocytes.

	HIIT		MICT		CTL	
	Pre	Post	Pre	Post	Pre	Post
Phospho-AMPK (mean fluorescence intensity)						
**Rt**	73.5 ± 3.1	83.4 ± 3.5	85.2 ± 3.8	87.1 ± 4.3	81.3 ± 3.2	80.1 ± 4.0
**HE**	113.8 ± 2.8*	95.5 ± 4.2	117.4 ± 4.9*	97.0 ± 5.1	119.3 ± 4.0*	117.0 ± 4.9*
SIRT (mean fluorescence intensity)						
**Rt**	22.6 ± 3.4	25.2 ± 3.0	23.4 ± 2.2	28.4 ± 3.0	23.9 ± 3.2	25.4 ± 3.2
**HE**	26.3 ± 3.7	25.8 ± 2.3	26.2 ± 4.6	25.6 ± 3.1	27.2 ± 3.5	29.6 ± 3.3
PCG-1α (mean fluorescence intensity)						
**Rt**	29.2 ± 4.7	26.0 ± 3.9	27.4 ± 2.7	31.1 ± 4.5	29.3 ± 2.5	30.2 ± 4.2
**HE**	32.1 ± 4.9	28.3 ± 3.5	33.8 ± 3.8	26.0 ± 2.1	33.2 ± 3.5	33.0 ± 2.3
NRF-1 (mean fluorescence intensity)						
**Rt**	65.0 ± 5.9	66.2 ± 6.1	65.1 ± 3.2	70.4 ± 6.7	69.1 ± 3.5	67.4 ± 6.0
**HE**	68.7 ± 5.4	78.3 ± 5.3	68.5 ± 4.6	73.7 ± 7.3	67.5 ± 4.2	70.5 ± 5.3
Tfam (mean fluorescence intensity)						
**Rt**	41.5 ± 4.1	45.1 ± 4.8	41.4 ± 5.9	45.2 ± 4.2	43.4 ± 5.0	47.2 ± 4.5
**HE**	46.8 ± 3.2	52.1 ± 5.4	47.5 ± 4.0	53.2 ± 5.8	46.7 ± 4.8	53.0 ± 5.1
Mitochondrial biogenesis (ratio of Complex IV to II)						
**Rt**	0.45 ± 0.07	0.46 ± 0.09	0.48 ± 0.05	0.50 ± 0.07	0.45 ± 0.05	0.49 ± 0.07
**HE**	0.50 ± 0.08	0.48 ± 0.08	0.45 ± 0.09	0.54 ± 0.07	0.55 ± 0.07	0.54 ± 0.08

Values were mean ± SE. **HIIT**, high-intensity interval training group; **MICT**, moderate-intensity continuous training group; **CTL**, control group; **Pre**, pre-intervention; **Post**, post-intervention; **Rt**, resting; **HE**, hypoxic exercise test. *P < 0.05, **Rt** vs. **HE**.

**Table 4:** The effects of interval and continuous exercise regimens on plasma catecholamine concentrations.

	HIIT		MICT		CTL	
	Pre	Post	Pre	Post	Pre	Post
Norepinephrine (pg/ml)						
**Rt**	255 ± 42	205 ± 22^+^	267 ± 40	245 ± 32	259 ± 34	267 ± 43
**HE**	961 ± 87*	421 ± 67*^+^	953 ± 92*	621 ± 79*^+^	993 ± 97*	1007 ± 109*
Epinephrine (pg/ml)						
**Rt**	76 ± 9	52 ± 7^+^	82 ± 10	64 ± 9	79 ± 8	81 ± 9
**HE**	125 ± 17*	88 ± 10*^+^	132 ± 16*	106 ± 11*^+^	121 ± 15*	126 ± 13*

Values were mean ± SE. **HIIT**, high-intensity interval training group; **MICT**, moderate-intensity continuous training group; **CTL**, control group; **Pre**, pre-intervention; **Post**, post-intervention; **Rt**, resting; **HE**, hypoxic exercise test. ^*^P < 0.05, **Rt** vs. **HE**, ^+^*P* < 0.05, **Pre** vs. **Post**.
